# MRI markers of neuroinflammation in untreated patients with subclinical generalized anxiety disorder

**DOI:** 10.1007/s00702-025-03014-x

**Published:** 2025-09-11

**Authors:** Balázs Barkó, Oguz Kelemen, Szabolcs Kéri

**Affiliations:** 1 Sárospatak College, Sztárai Institute, University of Tokaj, Eötvöst str. 7, Sárospatak, 3944 Hungary; 2 Department of Behavioral Science, Albert Szent-Györgyi Medical School, University of Szeged, Szeged, Hungary; 3 Department of Psychiatry, Bács-Kiskun County Hospital, Kecskemét, Hungary; 4 Department of Physiology, Albert Szent-Györgyi Medical School, University of Szeged, Szeged, Hungary

**Keywords:** Subclinical generalized anxiety disorder, Neuroinflammation, Amygdala, Hippocampus

## Abstract

Generalized Anxiety Disorder (GAD) is characterized by excessive worry and physical symptoms of prolonged anxiety. Patients with subclinical GAD-states (sub-GAD) do not fulfill the diagnostic criteria of GAD, but they often show a disease burden similar to GAD, and the subclinical state may turn into a full syndrome. Neuroinflammation may contribute to changes in brain structures in sub-GAD, but direct evidence remains lacking. We investigated 73 newly recruited sub-GAD patients who had never received pharmacological or psychological treatment and 64 matched non-clinical individuals. We utilized magnetic resonance imaging (MRI) to assess putative neuroinflammatory markers (DBSI-RF, diffusion-based spectral imaging-based restricted fraction) in the hippocampus, amygdala, and neocortex. The patients completed the Hamilton Anxiety Rating Scale (HAM-A), the Generalized Anxiety Disorder 7-item scale (GAD-7), and the Beck Depression Inventory-II (BDI-II). Compared to controls, sub-GAD individuals had significantly elevated inflammatory MRI markers in the amygdala but not in the hippocampus and neocortex. The DBSI-RF values correlated with the severity of anxiety (HAM-A and GAD-7 scores), but not with BDI-II. These findings suggest that neuroinflammation in the amygdala may play a critical role in the development of anxiety in sub-GAD.

## Introduction

Generalized Anxiety Disorder (GAD) is defined by persistent, overwhelming worry that is difficult to control, along with symptoms such as restlessness, both physical and mental fatigue, trouble focusing, irritability, muscle tightness, and sleep disturbances (American Psychiatric Association 2013). In the United States, 3.1% of the population is diagnosed with GAD each year, making it one of the most prevalent anxiety disorders (Kessler et al. 2012).

Recent studies examined the impact of neuroinflammation on the pathophysiology of GAD (Renna et al. 2018; Milaneschi et al. 2021; Glaus et al. 2018). Neuroinflammation associated with negative emotional states leads to dysregulation of the brain’s immune response, marked by the release of pro-inflammatory cytokines that impair neuronal information processing and metabolism (Rosenblat et al. 2014; Fries et al. 2023; Valiati et al. 2023). Consequently, research consistently shows higher levels of C-reactive protein (CRP), interleukin-6 (IL-6), and tumor necrosis factor-alpha (TNF-α) in GAD (Costello et al. 2019; Michopoulos et al. 2017; Tang et al. 2018). Additionally, elevated CRP and IL-6 levels correlate with specific anxiety symptoms like irritability and excessive worrying (Milaneschi et al. 2021).

Inflammation influences brain areas associated with mood regulation and fear, including the amygdala, hippocampus, insula, and anterior cingulate cortex, which may play a role in anxiety (Rosenblat et al. 2014; Goldsmith et al. 2023; Felger and Treadway 2017). The amygdala and hippocampus are crucial for emotion regulation, memory formation, and stress responses associated with GAD symptoms (Izquierdo et al. 2016; Lai 2020; Rezaei et al. 2023). The amygdala is often overactive in GAD, which is associated with increased emotional reactions and anxiety (Rauch et al. 2003). Furthermore, inflammation in the amygdala heightens its sensitivity to stressors, resulting in increased anxiety and emotional instability (Felger and Treadway 2017).

Current progress in magnetic resonance imaging (MRI) techniques enables the detection of potential indirect signs of neuroinflammation. The DBSI-RF (diffusion-basis spectral imaging-based restricted fraction) method identifies changes in the diffusion characteristics of water surrounding nerve fibers, signaling axonal injury, demyelination, and inflammation (Wang et al. 2011). The RF component quantifies the fraction of water molecules whose displacements over the diffusion-encoding interval remain spatially confined, typically because their paths are bounded by cell membranes or other subcellular structures. This means that water moving within densely packed cellular or subcellular spaces (e.g., tightly apposed axons, inflammatory cell infiltrates, or tumorous cellular regions) contributes disproportionately to the restricted compartment. As such, RF provides a direct, quantitative proxy for tissue cellularity and membrane integrity, enabling more specific delineation of pathological processes, such as inflammation, demyelination, or neoplasia, than conventional diffusion‐tensor metrics alone (Wang et al. 2014; Sun et al. 2020).

Although increased RF is not a direct and specific marker of neuroinflammation, multiple lines of evidence from basic research suggest its validity. In the rat axotomy model, the extra-axonal RF rose sharply after unilateral dorsal root transection and showed a strong correlation with microglial density on histology (Taquet et al. 2019). In mouse experimental autoimmune encephalomyelitis, RF was significantly elevated during disease onset and peak, with its spatial pattern co-localizing with histological markers of microglial infiltration and tissue cellularity (Wang et al. 2014). Finally, in traumatic optic neuropathy in mice, optic nerves showed elevated RF, reflecting infiltrating or proliferating cells in injured tissue (Yang et al. 2023).

DBSI-RF findings from human participants align with post-mortem cellular markers of neuroinflammatory disorders such as multiple sclerosis, obesity, and Alzheimer’s disease (Cross and Song 2017; Samara et al. 2019; Wang et al. 2019; Sun et al. 2020). Additionally, in individuals with depressive symptoms, heightened CRP levels were linked to increased RF (Zhang et al. 2023; Kéri and Kelemen 2024). Results revealed that effective psychotherapy reduces RF in the amygdala of patients with GAD (Kéri et al. 2024). The diagnostic criteria for Generalised Anxiety Disorder (GAD) have become more stringent in recent decades, and many patients with high anxiety levels who do not meet the GAD criteria may be excluded from effective treatment. The subclinical GAD states (sub-GAD) often present a similar disease burden to GAD and frequently progress to the full syndromal disorder (Diefenbach et al. 2003; Haller et al. 2014; Volz et al. 2022). In adolescents, higher levels of subclinical anxiety are associated with lower cortical thickness (Taylor et al. 2021). In addition, higher anxiety may result in poorer planning performance in non-clinical individuals (Unterrainer et al. 2018).

The primary objective of this study is to determine whether neuroinflammation can be detected in sub-GAD by using MRI DSBI-RF in the amygdala and hippocampus. The secondary objective was to determine the relationship between these putative neuroinflammatory markers and anxiety symptoms. Given that in our previous study treatment selectively decreased RF in the amygdala in GAD (Kéri et al. 2024), we hypothesized that increased RF in the amygdala is associated with higher anxiety in sub-GAD.

## Methods

### Participants

The individuals with sub-GAD (*n* = 74) were recruited from outpatient psychiatric clinics, general practitioners, and through online advertisements (National Psychiatric Center, Budapest; University of Szeged, Hungary). We administered the structured clinical interview for DSM-5 (Diagnosis and Statistical Manual of Mental Disorders – 5) for all participants (First et al. 2016). The inclusion criteria were as follows: the presence of DSM-5 items of GAD that did not reach the diagnostic threshold (diagnosis: Other Specified Anxiety Disorder - subthreshold GAD); age between 18 and 65 years; no mental disorders (e.g., major depressive disorder, bipolar disorder, psychotic disorders); not receiving any form of psychotherapy or psychotropic medications. The exclusion criteria included neurological disorders or head injury, substance use within the past six months, pregnancy or breastfeeding, using anti-inflammatory drugs, and any general contraindications to MRI.

We also included 64 control participants who were matched to the sub-GAD individuals for age, sex, education, and potential confounding variables affecting inflammation (nicotine, caffeine, and alcohol intake, contraception use, body mass index (BMI), chronic diseases, and working night shifts) (Narvaez Linares et al. 2020) (Table 1).

The study was conducted following the Declaration of Helsinki and approved by the National Medical Research Council (ETT-TUKEB 18814, Budapest, Hungary). Written Informed consent was obtained from all subjects involved in the study.

**Table 1:** Demographics and clinical characteristics

	Subclinical Generalized Anxiety Disorder (*n* = 74)	Control individuals (*n* = 64)	t/χ²	*p*
Age (years)	36.6 ± 12.4	36.7 ± 12.8	0.06	0.96
Education (years)	11.6 ± 3.1	12.3 ± 3.1	1.43	0.16
Sex (male/female)	30/44	24/40	0.13	0.72
Body Mass Index (BMI)	26.9 ± 8.4	26.4 ± 7.9	− 0.35	0.73
Hamilton Anxiety Rating Scale	22.3 ± 4.9	−		
Generalized Anxiety Disorder Scale-7	15.3 ± 4.4	−		
Beck depression inventory-II	11.6 ± 5.5	−		

Data are mean ± standard deviation except the sex ratio. Age, education, and BMI were compared with two-tailed *t* tests, whereas sex ratio was comapred with chi-square test. There were no significant differences between the two groups (*p*s > 0.1)

## Clinical assessment

We used the structured clinical interview for DSM-5 (First et al. 2016). Anxiety symptoms were evaluated using the Hamilton Anxiety Rating Scale (HAM-A) (Maier et al. 1988) and the Generalized Anxiety Disorder 7-item scale (GAD-7) (Spitzer et al. 2006). Depressive symptoms were assessed with the Beck Depression Inventory-II (BDI-II) (Wang and Gorenstein 2013).

The HAM-A is a semistructured interview (14 items, each rated from 0 (not present) to 4 (severe), with a total score of 0–56). Scoring ranges: 0–17 - mild anxiety, 18–24 - moderate anxiety, and 25–30 – severe anxiety (Maier et al. 1988). The Generalized Anxiety Disorder 7-item (GAD-7) scale is a self-report questionnaire (7 items, each rated from 0 (not at all) to 3 (nearly every day), with a score range of 0–21). The severity of anxiety is classified as minimal (0–4), mild (5–9), moderate (10–14), and severe (15–21) (Spitzer et al. 2006). The BDI-II is also a self-report questionnaire, consisting of 21 items, each with a rating scale of 0 to 3. The total score varies from 0 to 63, with classifications as follows: minimal (0–13), mild (14–19), moderate (20–28), and severe (29–63). (Wang and Gorenstein 2013).

## Magnetic resonance imaging (MRI)

Diffusion-weighted imaging (DWI) and T1-weighted structural scans were obtained in accordance with the United Kingdom (UK) Biobank protocol. Images were processed with FreeSurfer v7.4.1 (Fischl 2012). The scanning parameters were as follows: Philips Achieva 3 T scanner, MPRAGE (magnetization-prepared rapid acquisition gradient echo), 3D sagittal acquisition, FOV (square field of view) = 5256 mm, 1 × 1 × 1 mm^3^, TI = 5900 ms, TE (shortest) = 3.16, flip angle: 9 degrees, no fat suppression, full k space, no averages, acquisition time: 6 min and 50 s, acceleration factor: 2. Two b = 0 s/mm² volumes were acquired, one with anterior-posterior and one with posterior-anterior phase-encoding, for use in distortion correction and as the reference signal for the restricted-fraction fitting.

For the DWI data, we used a multi-shell approach (b1 = 1000 s/mm^2^, b2 = 2000 s/mm^2^, 2 × 2 × 2 mm^3^, 50 diffusion encoding directions for each shell). During DWI preprocessing, we applied corrections for head motion, outlier slices, and gradient distortion, and utilized eddy currents (Alfaro-Almagro et al. 2018; Zhang et al. 2023).

Head motion was quantified as framewise displacement (FD) calculated from the realignment parameters. We adopted FD > 1.5 mm per volume as an exclusion criterion. The mean FD was 0.67 mm (*SD* = 0.34) in the sub-GAD group, and 0.70 mm (*SD* = 0.40) in the control group (*p* > 0.2). No individual volume showed an FD jump from the preceding frame of more than 1.5 mm.

Putative neuroinflammatory changes were quantified using RF from DWI data (Wang et al. 2011, 2019). We focused on the hippocampus and amygdala utilizing FreeSurfer’s regions of interest (ROIs). From these ROIs, we extracted DBSI-RF (Fischl et al. 2002; Zhang et al. 2023; Jenkinson et al. 2012). The RF values for the left and right sides were averaged due to the high left-right correlations (*r* >0.8). The whole gray matter of the cortex served as a control ROI (Zhang et al. 2001, 2023).

### Data analysis

We used STATISTICA 13.1 (StatSoft) for data analysis. Following descriptive statistics and normality testing (Kolmogorov-Smirnov test), analysis of variance (ANOVA) was performed on the MRI RF values. The between-subjects factor was the experimental group (sub-GAD vs. controls), and the within-subjects factors was the brain regions (amygdala, hippocampus, and neocortex). The ANOVA effect size values were determined (partial eta-squared). Tukey’s honestly significant difference (HSD) tests were used for post-hoc comparisons. We also investigated the relationship between MRI RF values and clinical symptoms as measured with HAM-A, GAD-7, and BDI-II scores. We conducted multiple regression analyses to test these relationships and calculated Pearson’s correlation coefficients between RF values and clinical scale scores. The pre-corrected level of statistical significance was set at alpha < 0.05.

## Results

### MRI neuroinflammation markers

**Fig. 1:**
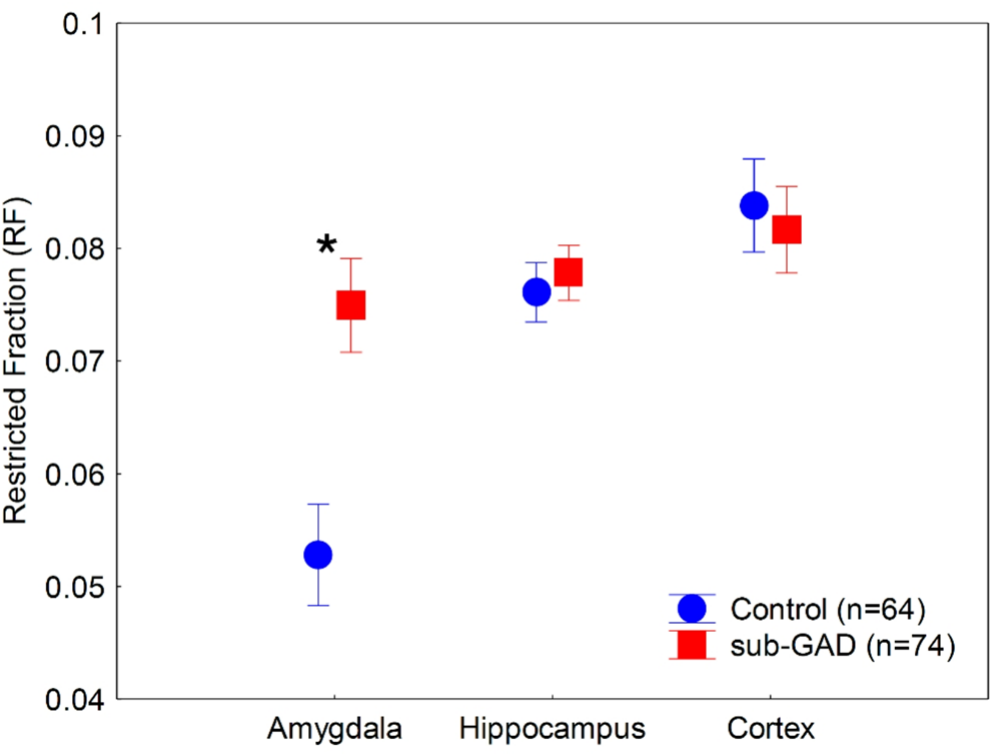
MRI-restricted fraction (RF) values in patients with Generalized Anxiety Disorder (GAD) and matched controls. Data are presented as means, and error bars indicate 95% confidence intervals. * *p* < 0.001 (Tukey’s HSD test)

The ANOVA conducted on the RF values revealed significant main effects of group (sub-GAD vs. controls) (*F*(1,136) = 19.87, *p* < 0.001, *ƞ*^*2*^ = 0.13) and brain regions (amygdala, hippocampus, and cortex) (*F*(2,272) = 56.72, *p* < 0.001, *ƞ*^*2*^ = 0.29). The two-way interaction between the group and brain regions was also significant (*F*(2,272) = 25.83, *p* < 0.001, *ƞ*^*2*^ = 0.16). As shown in Fig. 1, patients with sub-GAD exhibited elevated RF values in the amygdala, but not in the hippocampus or cortex, compared to the control group (*p* < 0.001, Tukey’s HSD test).

## Relationship between clinical symptoms and MRI neuroinflammation markers

**Fig. 2:**
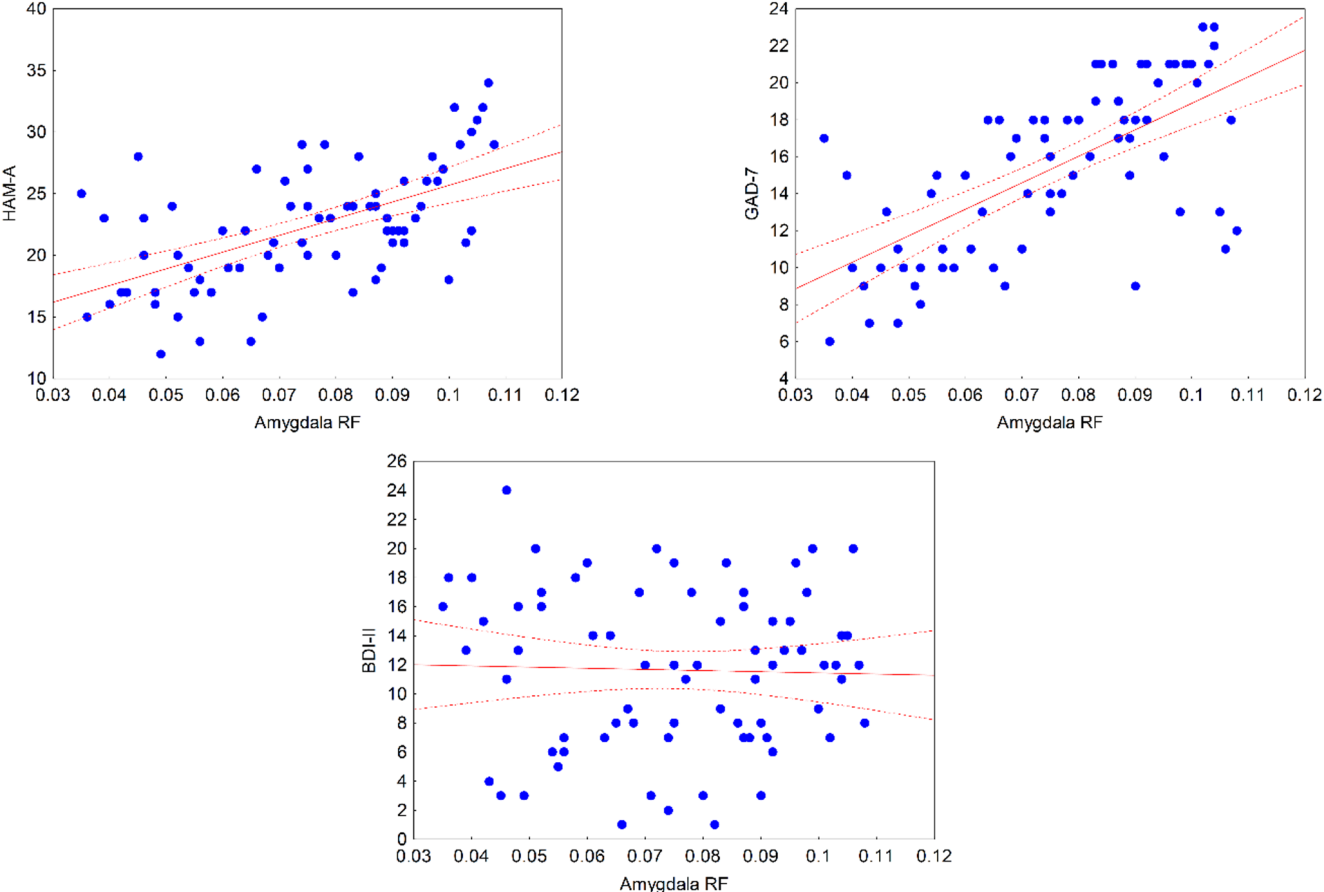
Correlations between amygdala restricted fraction (RF) values and Hamilton Anxiety Rating Scale (HAM-A) (*r* = 0.58, *p* < 0.001), Generalized Anxiety Disorder 7-item scale (GAD-7) (*r* = 0.67, *p* < 0.001), and Beck Depression Inventory-II (BDI-II) values (*r* = −0.030, *p* = 0.8)

The HAM-A scores showed a positive correlation with amygdalar RF values (*r* = 0.58, *p* < 0.001) and a weak correlation with RF values in the hippocampus (*r* = 0.30, *p* = 0.01). The correlation was not significant in the cortex (*r* = 0.02, *p* = 0.88). Similar to the HAM-A scores, the GAD-7 scores showed a positive correlation with amygdalar RF values (*r* = 0.67, *p* < 0.001) and a weak correlation with RF values in the hippocampus (*r* = 0.27, *p* = 0.02). The correlation was not significant in the cortex (*r* = 0.02, *p* = 0.87). We did not find significant correlations between the RF values and BDI-II scores (*r*s = −0.03 to 0.2, *p*s > 0.1). Following Bonferroni correction, only the correlation between the HAM-A/GAD7 scores and the amygdalar RF values remained significant (*p* < 0.005) (Fig. 2).

**Fig. 3:**
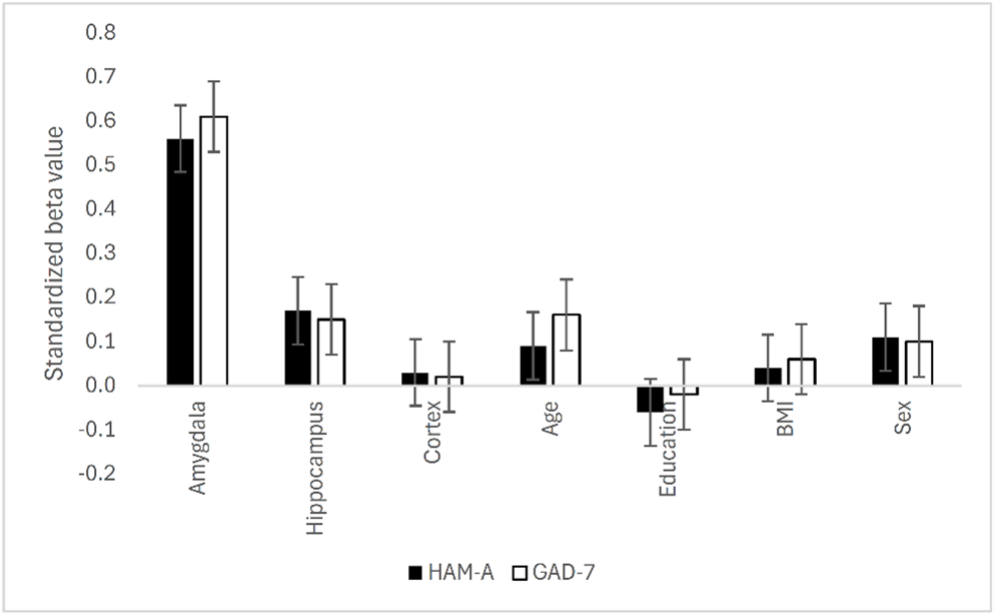
Standardized beta coefficients from the regression analyses. Error bars indicate standard error. HAM-A: Hamilton Anxiety Rating Scale, GAD-7: Generalized Anxiety Disorder 7-item scale

Multiple regression analyses were consistent with the correlation analyses. The HAM-A scores were predicted solely by amygdalar RF values after adjusting for sex, age, education, and BMI (β* = 0.56, *R²* = 0.15, *t*(67) = 5.33, *p* < 0.001). Similar results appeared for the GAD7 scores (β* = 0.61, *R²* = 0.15, *t*(67) = 6.32, *p* < 0.001). The standardized beta coefficients from the regression analyses are shown in Fig. 3.

## Discussion

We used a novel diffusion-basis spectral MRI technique to investigate the role of neuroinflammation in sub-GAD. We focused on a cohort of newly diagnosed and untreated sub-GAD individuals. In this way, we minimized the confounding effects of prior treatments. We also explored the relationship between regional neuroinflammation and the severity of clinical symptoms. We found elevated RF values in the amygdala of sub-GAD individuals compared to controls, which suggests that inflammation in this emotion-processing region may contribute to the pathophysiology of sub-GAD. Moreover, RF values correlated with the severity of anxiety but not with the level of depressive symptoms. There were no similar changes in the hippocampus and cortex, which indicates a region-specificity in sub-GAD.

MRI DBSI-RF has been previously validated in conditions such as multiple sclerosis, obesity, and Alzheimer’s disease, where neuroinflammatory processes have been proven (Cross and Song 2017; Samara et al. 2019; Wang et al. 2011, 2019). We extended its application to psychiatric disorders, offering a potential new biomarker for anxiety disorders. The selection of specific brain regions (amygdala, hippocampus, and neocortex) was motivated by existing neuroanatomical models of anxiety (Andres et al. 2025; Hennings et al. 2022). The role of the amygdala in fear and emotional responses further supports the notion that its alteration may be a key biomarker for anxiety disorders (Kenwood et al. 2022).

It must be noted that increased RF may be an indicator of axonal loss, demyelination, edema, or other changes in the tissue organisation. However, axonal loss or demyelination is not likely in sub-GAD, and the most plausible explanation for increased RF is elevated extracellular water associated with mild, stress-related neuroinflammation.

The findings of this study contribute to an emerging body of literature that links neuroinflammation with psychiatric disorders and extend these findings to subclinical states. Previous research has consistently demonstrated that individuals with GAD and other anxiety disorders exhibit elevated levels of peripheral inflammatory markers (e.g., CRP, IL-6, TNF-α) (Michopoulos et al. 2017; Costello et al. 2019; Tang et al. 2018). These peripheral changes have been associated with symptom severity and proposed to affect brain functions through various mechanisms, including the modulation of neurotransmitter systems and neural connectivity in the reward circuit and fear detection system (Bekhbat et al. 2022; Hu et al. 2022).

In this context, our study extends previous work by providing in vivo evidence of localized inflammatory changes in the amygdala, characterized at least by increased extracellular water content. Evidence suggests hyperactivity in the amygdala in response to stress and fear-inducing stimuli (Ashworth et al. 2021). The current results indicate that neuroinflammation might exacerbate this hyperactivity, potentially leading to heightened emotional reactivity and anxiety. Moreover, the lack of inflammatory changes in the hippocampus contrasts with the findings of studies investigating major depressive disorder, where hippocampal neuroinflammation has been detected (Kaszás et al. 2025). This divergence may underscore a unique profile in GAD relative to mood disorders.

In rodent models of repeated stress, one can observe an increase in amygdala RF, microglia activation, and higher in vivo firing rates of basolateral amygdala neurons. Pharmacological blockade of microglial activation prevents neuronal hyper-firing and anxiety-like behaviors (Munshi et al. 2020). Second, stress up-regulates IL-1β, IL-6, and TNF-α in the basolateral amygdala; these cytokines have been shown to increase c-Fos expression (a marker of neuronal activation) and to shift the balance toward enhanced glutamatergic transmission and/or reduced GABA-ergic inhibition (Hu et al. 2022). Finally, reactive astrocytes can down-regulate glutamate transporters (GLT-1) and K⁺ buffering, so neurotransmitter clearance is impaired and extracellular glutamate/K⁺ accumulate, leading to neuronal hyper-excitability (Verkhratsky et al. 2023).

From a clinical perspective, the demonstration of a neuroinflammatory component in sub-GAD may have important therapeutic implications. If neuroinflammation contributes to the pathogenesis of anxiety even in diagnostically subclinical cases, interventions that target inflammatory pathways may offer an effective treatment. Anti-inflammatory agents, already under investigation for mood and anxiety disorders, could potentially also be used in sub-GAD (Sah and Singewald 2025). Moreover, DBSI-RF and similar imaging biomarkers could be used to monitor treatment response and disease progression. This allows clinicians to tailor interventions based on neurobiological changes rather than solely on symptomatic improvements.

The present study has limitations. The study’s cross-sectional nature limits causal interpretations. While elevated neuroinflammation in the amygdala is associated with increased anxiety severity, it remains unclear whether inflammation is a driving force behind sub-GAD or a downstream effect of chronic anxiety.

Although DBSI-RF is a promising marker for neuroinflammation, its specificity requires further investigation. Changes in the restricted fraction may also reflect other pathological processes, such as axonal injury or demyelination with different origins (van der Weijden et al. 2023). Without direct measures of inflammatory cytokines or additional imaging modalities (e.g., PET imaging with microglial markers), interpreting DBSI-RF as a marker of inflammation should be approached cautiously.

While significant alterations were detected in the amygdala, no differences were observed in the hippocampus or neocortex. This regional specificity raises questions about the generalizability of neuroinflammatory changes in sub-GAD. It may be that the amygdala is uniquely vulnerable to inflammatory processes in the context of anxiety, or it could reflect limitations in the spatial resolution or sensitivity of the imaging protocol used for smaller or more heterogeneous brain regions.

## Conclusions

Our results contribute to the understanding of anxiety spectrum disorders by demonstrating that neuroinflammatory markers, as measured by DBSI-RF, are elevated in the amygdala of untreated individuals experiencing anxiety. It is essential to note that the methodology, including the selection of unmedicated individuals with subthreshold symptoms and the use of advanced imaging techniques, underpins the reliability of these findings. However, limitations such as the cross-sectional design, potential nonspecificity of the imaging marker, and absence of peripheral inflammatory measures warrant cautious interpretation and necessitate further studies.

## Data Availability

The data of this study are available on request from the corresponding author.

## References

[CR1] Alfaro-Almagro F, Jenkinson M, Bangerter NK, Andersson JLR, Griffanti L, Douaud G, Sotiropoulos SN, Jbabdi S, Hernandez-Fernandez M, Vallee E, Vidaurre D, Webster M, McCarthy P, Rorden C, Daducci A, Alexander DC, Zhang H, Dragonu I, Matthews PM, Miller KL, Smith SM (2018) Image processing and quality control for the first 10 000 brain imaging datasets from UK biobank. Neuroimage 166:400–424. 10.1016/j.neuroimage.2017.10.034 29079522 10.1016/j.neuroimage.2017.10.034 PMC5770339

[CR2] Andres E, Meyer B, Yuen KSL, Kalisch R (2025) Current state of the neuroscience of fear extinction and its relevance to anxiety disorders. Curr Top Behav Neurosci. 10.1007/7854_2024_555 39747796 10.1007/7854_2024_555

[CR3] Ashworth E, Brooks SJ, Schiöth HB (2021) Neural activation of anxiety and depression in children and young people: a systematic meta-analysis of fMRI studies. Psychiatry Res Neuroimaging 311:111272. 10.1016/j.pscychresns.2021.111272 33725661 10.1016/j.pscychresns.2021.111272

[CR4] American Psychiatric Association (2013) Diagnostic and statistical manual of mental disorders. (ed) Diagnostic and statistical manual of mental disorders, 5th edn. American Psychiatric, Washington, American Psychiatric Press

[CR5] Bekhbat M, Treadway MT, Felger JC (2022) Inflammation as a pathophysiologic pathway to anhedonia: mechanisms and therapeutic implications. Curr Top Behav Neurosci 58:397–419. 10.1007/7854_2021_294 34971449 10.1007/7854_2021_294

[CR6] Costello H, Gould RL, Abrol E, Howard R (2019) Systematic review and meta-analysis of the association between peripheral inflammatory cytokines and generalised anxiety disorder. BMJ Open 9:e027925. 10.1136/bmjopen-2018-027925 31326932 10.1136/bmjopen-2018-027925 PMC6661660

[CR7] Cross AH, Song SK (2017) A new imaging modality to non-invasively assess multiple sclerosis pathology. J Neuroimmunol 304:81–85. 10.1016/j.jneuroim.2016.10.002 27773433 10.1016/j.jneuroim.2016.10.002 PMC5316501

[CR8] Diefenbach GJ, Hopko DR, Feigon S, Stanley MA, Novy DM, Beck JG, Averill PM (2003) Minor GAD’: characteristics of subsyndromal GAD in older adults. Behav Res Ther 41:481–487. 10.1016/S0005-7967(02)00130-4 12643969 10.1016/s0005-7967(02)00130-4

[CR9] Felger JC, Treadway MT (2017) Inflammation effects on motivation and motor activity: role of dopamine. Neuropsychopharmacology 42:216–241. 10.1038/npp.2016.143 27480574 10.1038/npp.2016.143 PMC5143486

[CR10] First MB, Williams JBW, Karg RS, Spitzer RL (2016) Structured clinical interview for DSM-5 Disorders—Clinician version (SCID-5-CV). American Psychiatric Association Publishing, Washington

[CR11] Fischl B (2012) FreeSurfer. Neuroimage 62:774–781. 10.1016/j.neuroimage.2012.01.021 22248573 10.1016/j.neuroimage.2012.01.021 PMC3685476

[CR12] Fischl B, Salat DH, Busa E, Albert M, Dieterich M, Haselgrove C, van der Kouwe A, Killiany R, Kennedy D, Klaveness S, Montillo A, Makris N, Rosen B, Dale AM (2002) Whole brain segmentation: automated labeling of neuroanatomical structures in the human brain. Neuron 33:341–355. 10.1016/S0896-6273(02)00569-X 11832223 10.1016/s0896-6273(02)00569-x

[CR13] Fries GR, Saldana VA, Finnstein J, Rein T (2023) Molecular pathways of major depressive disorder converge on the synapse. Mol Psychiatry 28:284–297. 10.1038/s41380-022-01806-1 36203007 10.1038/s41380-022-01806-1 PMC9540059

[CR14] Glaus J, von Känel R, Lasserre AM, Strippoli MF, Vandeleur CL, Castelao E, Gholam-Rezaee M, Marangoni C, Wagner EN, Marques-Vidal P, Waeber G, Vollenweider P, Preisig M, Merikangas KR (2018) The bidirectional relationship between anxiety disorders and Circulating levels of inflammatory markers: results from a large longitudinal population-based study. Depress Anxiety 35:360–371. 10.1002/da.22710 29244900 10.1002/da.22710

[CR15] Goldsmith DR, Bekhbat M, Mehta ND, Felger JC (2023) Inflammation-related functional and structural dysconnectivity as a pathway to psychopathology. Biol Psychiatry 93:405–418. 10.1016/j.biopsych.2022.11.003 36725140 10.1016/j.biopsych.2022.11.003 PMC9895884

[CR16] Haller H, Cramer H, Lauche R, Gass F, Dobos GJ (2014) The prevalence and burden of subthreshold generalized anxiety disorder: a systematic review. BMC Psychiatry 14:128. 10.1186/1471-244X-14-128 24886240 10.1186/1471-244X-14-128 PMC4048364

[CR17] Hennings AC, Cooper SE, Lewis-Peacock JA, Dunsmoor JE (2022) Pattern analysis of neuroimaging data reveals novel insights on threat learning and extinction in humans. Neurosci Biobehav Rev 142:104918. 10.1016/j.neubiorev.2022.104918 36257347 10.1016/j.neubiorev.2022.104918 PMC11163873

[CR18] Hu P, Lu Y, Pan BX, Zhang WH (2022) New insights into the pivotal role of the amygdala in inflammation-related depression and anxiety disorder. Int J Mol Sci. 10.3390/ijms231911076 36232376 10.3390/ijms231911076 PMC9570160

[CR19] Izquierdo I, Furini CR, Myskiw JC (2016) Fear memory. Physiol Rev 96:695–750. 10.1152/physrev.00018.2015 26983799 10.1152/physrev.00018.2015

[CR20] Jenkinson M, Beckmann CF, Behrens TEJ, Woolrich MW, Smith SM (2012) FSL Neuroimage 62:782–790. 10.1016/j.neuroimage.2011.09.015 21979382 10.1016/j.neuroimage.2011.09.015

[CR21] Kaszás A, Kelemen O, Kéri S (2025) Magnetic resonance imaging signatures of neuroinflammation in major depressive disorder with religious and spiritual problems. Sci Rep 15:5407. 10.1038/s41598-025-89581-1 39948408 10.1038/s41598-025-89581-1 PMC11825903

[CR22] Kenwood MM, Kalin NH, Barbas H (2022) The prefrontal cortex, pathological anxiety, and anxiety disorders. Neuropsychopharmacology 47:260–275. 10.1038/s41386-021-01109-z 34400783 10.1038/s41386-021-01109-z PMC8617307

[CR23] Kéri S, Kelemen O (2024) Signatures of neuroinflammation in the hippocampus and amygdala in individuals with religious or spiritual problem. Relig Brain Behav:1–13. 10.1080/2153599X.2024.2349784

[CR24] Kéri S, Kancsev A, Kelemen O (2024) Algorithm-based modular psychotherapy alleviates brain inflammation in generalized anxiety disorder. Life. 10.3390/life14070887 39063640 10.3390/life14070887 PMC11278507

[CR25] Kessler RC, Avenevoli S, Costello J, Green JG, Gruber MJ, McLaughlin KA, Petukhova M, Sampson NA, Zaslavsky AM, Merikangas KR (2012) Severity of 12-month DSM-IV disorders in the National comorbidity survey replication adolescent supplement. Arch Gen Psychiatry 69:381–389. 10.1001/archgenpsychiatry.2011.1603 22474106 10.1001/archgenpsychiatry.2011.1603 PMC3522117

[CR26] Lai CH (2020) Task MRI-based functional brain network of anxiety. Adv Exp Med Biol 1191:3–20. 10.1007/978-981-32-9705-0_1 32002919 10.1007/978-981-32-9705-0_1

[CR27] Maier W, Buller R, Philipp M, Heuser I (1988) The Hamilton anxiety scale: reliability, validity and sensitivity to change in anxiety and depressive disorders. J Affect Disord 14:61–68. 10.1016/0165-0327(88)90072-9 2963053 10.1016/0165-0327(88)90072-9

[CR28] Michopoulos V, Powers A, Gillespie CF, Ressler KJ, Jovanovic T (2017) Inflammation in fear- and anxiety-based disorders: PTSD, GAD, and beyond. Neuropsychopharmacology 42:254–270. 10.1038/npp.2016.146 27510423 10.1038/npp.2016.146 PMC5143487

[CR29] Milaneschi Y, Kappelmann N, Ye Z, Lamers F, Moser S, Jones PB, Burgess S, Penninx B, Khandaker GM (2021) Association of inflammation with depression and anxiety: evidence for symptom-specificity and potential causality from UK biobank and NESDA cohorts. Mol Psychiatry 26:7393–7402. 10.1038/s41380-021-01188-w 34135474 10.1038/s41380-021-01188-w PMC8873022

[CR30] Munshi S, Loh MK, Ferrara N, DeJoseph MR, Ritger A, Padival M, Record MJ, Urban JH, Rosenkranz JA (2020) Repeated stress induces a pro-inflammatory state, increases amygdala neuronal and microglial activation, and causes anxiety in adult male rats. Brain Behav Immun 84:180–199. 10.1016/j.bbi.2019.11.023 31785394 10.1016/j.bbi.2019.11.023 PMC7010555

[CR31] Narvaez Linares NF, Charron V, Ouimet AJ, Labelle PR, Plamondon H (2020) A systematic review of the Trier social stress test methodology: issues in promoting study comparison and replicable research. Neurobiol Stress 13:100235. 10.1016/j.ynstr.2020.100235 33344691 10.1016/j.ynstr.2020.100235 PMC7739033

[CR32] Rauch SL, Shin LM, Wright CI (2003) Neuroimaging studies of amygdala function in anxiety disorders. Ann N Y Acad Sci 985:389–410. 10.1111/j.1749-6632.2003.tb07096.x 12724173 10.1111/j.1749-6632.2003.tb07096.x

[CR33] Renna ME, O’Toole MS, Spaeth PE, Lekander M, Mennin DS (2018) The association between anxiety, traumatic stress, and obsessive-compulsive disorders and chronic inflammation: a systematic review and meta-analysis. Depress Anxiety 35:1081–1094. 10.1002/da.22790 30199144 10.1002/da.22790

[CR34] Rezaei S, Gharepapagh E, Rashidi F, Cattarinussi G, Sanjari Moghaddam H, Di Camillo F, Schiena G, Sambataro F, Brambilla P, Delvecchio G (2023) Machine learning applied to functional magnetic resonance imaging in anxiety disorders. J Affect Disord 342:54–62. 10.1016/j.jad.2023.09.006 37683943 10.1016/j.jad.2023.09.006

[CR35] Rosenblat JD, Cha DS, Mansur RB, McIntyre RS (2014) Inflamed moods: a review of the interactions between inflammation and mood disorders. Prog Neuropsychopharmacol Biol Psychiatry 53:23–34. 10.1016/j.pnpbp.2014.01.013 24468642 10.1016/j.pnpbp.2014.01.013

[CR36] Sah A, Singewald N (2025) The (neuro)inflammatory system in anxiety disorders and PTSD: potential treatment targets. Pharmacol Ther 269:108825. 10.1016/j.pharmthera.2025.108825 39983845 10.1016/j.pharmthera.2025.108825

[CR37] Samara A, Murphy T, Strain J, Rutlin J, Sun P, Neyman O, Sreevalsan N, Shimony JS, Ances BM, Song SK, Hershey T, Eisenstein SA (2019) Neuroinflammation and white matter alterations in obesity assessed by diffusion basis spectrum imaging. Front Hum Neurosci 13:464. 10.3389/fnhum.2019.00464 31992978 10.3389/fnhum.2019.00464 PMC6971102

[CR38] Spitzer RL, Kroenke K, Williams JB, Löwe B (2006) A brief measure for assessing generalized anxiety disorder: the GAD-7. Arch Intern Med 166:1092–1097. 10.1001/archinte.166.10.1092 16717171 10.1001/archinte.166.10.1092

[CR39] Sun P, George A, Perantie DC, Trinkaus K, Ye Z, Naismith RT, Song S-K, Cross AH (2020) Diffusion basis spectrum imaging provides insights into MS pathology. Neurol Neuroimmunol Neuroinflamm 7:e655. 10.1212/NXI.0000000000000655 31871296 10.1212/NXI.0000000000000655 PMC7011117

[CR40] Tang Z, Ye G, Chen X, Pan M, Fu J, Fu T, Liu Q, Gao Z, Baldwin DS, Hou R (2018) Peripheral proinflammatory cytokines in Chinese patients with generalised anxiety disorder. J Affect Disord 225:593–598. 10.1016/j.jad.2017.08.082 28886500 10.1016/j.jad.2017.08.082

[CR41] Taquet M, Jankovski A, Rensonnet G, Jacobs D, des Rieux A, Macq B, Warfield SK, Scherrer B (2019) Extra-axonal restricted diffusion as an in-vivo marker of reactive microglia. Sci Rep 9:13874. 10.1038/s41598-019-50432-5 31554896 10.1038/s41598-019-50432-5 PMC6761095

[CR42] Taylor BK, Eastman JA, Frenzel MR, Embury CM, Wang Y-P, Stephen JM, Calhoun VD, Badura-Brack AS, Wilson TW (2021) Subclinical anxiety and posttraumatic stress influence cortical thinning during adolescence. J Am Acad Child Adolesc Psychiatry 60:1288–1299. 10.1016/j.jaac.2020.11.020 33383162 10.1016/j.jaac.2020.11.020 PMC8236497

[CR43] Unterrainer JM, Domschke K, Rahm B, Wiltink J, Schulz A, Pfeiffer N, Lackner KJ, Münzel T, Wild PS, Beutel M (2018) Subclinical levels of anxiety but not depression are associated with planning performance in a large population-based sample. Psychol Med 48:168–174. 10.1017/S0033291717002562 28874209 10.1017/S0033291717002562

[CR44] Valiati FE, Feiten JG, Géa LP, Silveira Júnior ÉM, Scotton E, Caldieraro MA, Salum GA, Kauer-Sant’Anna M (2023) Inflammation and damage-associated molecular patterns in major psychiatric disorders. Trends Psychiatry Psychother 45:e20220576. 10.47626/2237-6089-2022-0576 36527709 10.47626/2237-6089-2022-0576 PMC10640887

[CR45] Verkhratsky A, Butt A, Li B, Illes P, Zorec R, Semyanov A, Tang Y, Sofroniew MV (2023) Astrocytes in human central nervous system diseases: a frontier for new therapies. Signal Transduct Target Ther 8:396. 10.1038/s41392-023-01628-9 37828019 10.1038/s41392-023-01628-9 PMC10570367

[CR46] Volz HP, Saliger J, Kasper S, Möller HJ, Seifritz E (2022) Subsyndromal generalised anxiety disorder: operationalisation and epidemiology—a systematic literature survey. Int J Psychiatry Clin Pract 26:277–286. 10.1080/13651501.2021.1941120 34314295 10.1080/13651501.2021.1941120

[CR47] Wang YP, Gorenstein C (2013) Psychometric properties of the Beck depression Inventory-II: a comprehensive review. Braz J Psychiatry 35:416–431. 10.1590/1516-4446-2012-1048 24402217 10.1590/1516-4446-2012-1048

[CR48] Wang Y, Wang Q, Haldar JP, Yeh FC, Xie M, Sun P, Tu TW, Trinkaus K, Klein RS, Cross AH, Song SK (2011) Quantification of increased cellularity during inflammatory demyelination. Brain 134:3590–3601. 10.1093/brain/awr307 22171354 10.1093/brain/awr307 PMC3235568

[CR49] Wang X, Cusick MF, Wang Y, Sun P, Libbey JE, Trinkaus K, Fujinami RS, Song SK (2014) Diffusion basis spectrum imaging detects and distinguishes coexisting subclinical inflammation, demyelination and axonal injury in experimental autoimmune encephalomyelitis mice. NMR Biomed 27:843–852. 10.1002/nbm.3129 24816651 10.1002/nbm.3129 PMC4071074

[CR50] Wang Q, Wang Y, Liu J, Sutphen CL, Cruchaga C, Blazey T, Gordon BA, Su Y, Chen C, Shimony JS, Ances BM, Cairns NJ, Fagan AM, Morris JC, Benzinger TLS (2019) Quantification of white matter cellularity and damage in preclinical and early symptomatic Alzheimer’s disease. NeuroImage: Clinical 22:101767. 10.1016/j.nicl.2019.101767 30901713 10.1016/j.nicl.2019.101767 PMC6428957

[CR51] van der Weijden CWJ, Biondetti E, Gutmann IW, Dijkstra H, McKerchar R, de Paula Faria D, de Vries EFJ, Meilof JF, Dierckx R, Prevost VH, Rauscher A (2023) Quantitative myelin imaging with MRI and PET: an overview of techniques and their validation status. Brain 146:1243–1266. 10.1093/brain/awac436 36408715 10.1093/brain/awac436 PMC10115240

[CR52] Yang H-C, Lavadi RS, Sauerbeck AD, Wallendorf M, Kummer TT, Song S-K, Lin T-H (2023) Diffusion basis spectrum imaging detects subclinical traumatic optic neuropathy in a closed-head impact mouse model of traumatic brain injury. Front Neurol 14:126981. 10.3389/fneur.2023.126981 10.3389/fneur.2023.1269817 PMC10752006 38152638

[CR53] Zhang Y, Brady M, Smith S (2001) Segmentation of brain MR images through a hidden Markov random field model and the expectation-maximization algorithm. IEEE Trans Med Imaging 20:45–57. 10.1109/42.906424 11293691 10.1109/42.906424

[CR54] Zhang W, Rutlin J, Eisenstein SA, Wang Y, Barch DM, Hershey T, Bogdan R, Bijsterbosch JD (2023) Neuroinflammation in the amygdala is associated with recent depressive symptoms. Biol Psychiatry Cogn Neurosci Neuroimaging. 10.1016/j.bpsc.2023.04.011 37164312 10.1016/j.bpsc.2023.04.011 PMC13386403

